# Prenatal and postnatal bisphenol A exposure and social impairment in 4-year-old children

**DOI:** 10.1186/s12940-017-0289-2

**Published:** 2017-07-26

**Authors:** Youn-Hee Lim, Sanghyuk Bae, Bung-Nyun Kim, Choong Ho Shin, Young Ah Lee, Johanna Inhyang Kim, Yun-Chul Hong

**Affiliations:** 1 Institute of Environmental Medicine, Seoul National University Medical Research Center, Seoul, Republic of Korea; 2 Environmental Health Center, Seoul National University College of Medicine, Seoul, Republic of Korea; 3 Department of Preventive Medicine, Dankook University, Cheonan, Republic of Korea; 4 Division of Children and Adolescent Psychiatry, Department of Psychiatry, Seoul National University Hospital, Seoul, Republic of Korea; 5 Department of Pediatrics, Seoul National University Children’s Hospital, Seoul, Republic of Korea; 6 Department of Preventive Medicine, Seoul National University College of Medicine, Seoul, Republic of Korea

**Keywords:** Bisphenol A, Childhood exposure, Children’s environmental health, Prenatal exposure, Social impairment

## Abstract

**Background:**

Prenatal and postnatal exposure to bisphenol A (BPA) may affect early brain development. Rodent studies suggest that prenatal and postnatal neurodevelopmental toxicity from BPA exposure may manifest as social deficits in offspring. We investigated the association between prenatal and postnatal exposure to BPA and social impairments in a sample of 4-year-old children.

**Methods:**

We recruited second-trimester pregnant women between 2008 and 2011, and measured their creatinine-adjusted prenatal urine BPA levels. In 2014-2015, a subset of 4-year-old children born to these women underwent neurobehavioral assessment and physical examination. We collected urine and blood from the children and assessed social impairments, including deficits in social interaction, social communication, and other behavior patterns using the Korean version of the Social Communication Questionnaire (K-SCQ) (*n* = 304). We examined social impairments associated with prenatal exposure at mid-term pregnancy and postnatal exposure to BPA at 4 years of age, using linear and piecewise linear regression models.

**Results:**

The relationship between prenatal BPA exposure and social communication was non-linear and statistically significant at or above the flexion point for BPA levels of 3.0 μg/g creatinine in girls (58.4%, 95% confidence interval [CI], 6.5% to 135.8%). Each 2-fold increase in postnatal BPA exposure was significantly associated with an 11.8% (95% CI, 0.6% to 24.3%) increase in impairment in social communication in 4-year old girls, as indicated by the linear regression model.

**Conclusion:**

Prenatal and postnatal BPA exposure is associated with social impairment at 4 years of age, particularly in girls.

**Electronic supplementary material:**

The online version of this article (doi:10.1186/s12940-017-0289-2) contains supplementary material, which is available to authorized users.

## Background

Bisphenol A (BPA) is an organic synthetic compound used to make plastics and epoxy resins. Widespread use of BPA results in contamination in every individual [1–4]. In particular, BPA has been detected in the urine of pregnant women and children [5–11]. The health effects of prenatal and postnatal BPA exposure are reflected in children’s behavior [12–15], and as anxiety and depression [16]. However, few studies have examined social impairments associated with prenatal and postnatal exposure to BPA. Rodent studies indicate that exposure to low doses of BPA in utero have immediate and enduring transgenerational effects on social interactions and recognition [17, 18]. However, cohort studies examining prenatal exposure to BPA have not found significant associations with social impairments in children 4-9 years of age [19, 20].

Social impairment is defined as a lack of involvement in relationships with others. It typically occurs with neurological development problems such as autism spectrum disorders (ASDs). The European Union has expressed concern regarding data [21] indicating that exposure to endocrine disruptors may contribute to neurobehavioral deficits and disease, which cost more than €150 billion per year in Europe. In an effort to better control endocrine-disrupting chemicals, the European Food Safety Authority reduced the safety level of BPA from a combination of sources (e.g., diet, dust, cosmetics, and thermal paper) from 50 μg/kg of body weight/day to 4 μg/kg in 2015 [22]. However, uncertainty persists regarding the transgenerational effects of low-dose exposure to BPA on neurodevelopmental health, as assessed by toxicological testing [23]. Moreover, a non-monotonic dose-response relationship between low-dose endocrine disruptors and measures of behavioral and learning problems complicates our understanding of the biological effects of BPA [24–27]. In addition, sex- and time-specific effects on neurodevelopment or behavioral outcomes following BPA exposure have been reported in animal studies [28–32]; however, the traits that are most sensitive to BPA in humans have not been fully elucidated [12, 14–16]. Therefore, birth cohort studies are needed to confirm the time- and gender-dependent nature of BPA exposure by examining the dose-response relationship between prenatal and postnatal exposure to low-dose BPA and social impairment in girls and boys. In the present study, we used an ongoing cohort of 4-year-old children to examine social impairments associated with prenatal and postnatal exposure to BPA, using linear and non-linear regression models to examine overall and threshold BPA effects. In addition, we examined gender-specific effects on social impairment.

## Methods

### Population

The present study, the Environment and Development of Children (EDC) Study, is a prospective cohort study of the growth and development of children. The participants are children whose mothers participated in another study of birth outcomes, the Congenital Anomaly Study (CAS). The CAS cohort consisted of pregnant women who received prenatal care at 1 of 8 hospitals in the metropolitan areas of Seoul and Incheon, the Republic of Korea. The study enrolled 13,484 women during the second trimester of pregnancy and 11,085 of these women remained in the study until they gave birth between August 2008 and July 2011. At the time of enrollment, blood and urine samples were collected after more than 8 h of fasting and a questionnaire regarding demographics and lifestyle was administered by trained nurses. The CAS cohort included 115 children with congenital anomalies. After excluding mothers having children with congenital anomalies (*n* = 115) and those with invalid addresses (*n* = 218), 10,752 mothers were target participants for a new birth cohort comprising the EDC study (Additional file 1: Figure S1(a)).

We determined that a sample size of 610 (effect size 0.017 [33], alpha 0.05, power 0.90) would sufficiently examine the association between BPA exposure and children’s growth variables such as body mass index (BMI); we inflated the sample size to 645 children in order to allow for an ~5% drop-out rate. Between 2012 and 2015, we contacted 2085 mothers chosen randomly from the 10,752 target participants, until enrolling 645 mother-child pairs (615 mothers, including 30 multiple births) in the EDC study (response rate, 31%). We conducted follow-ups when the children were approximately 4 years of age, between March 2013 and December 2015 (Additional file 1: Figure S1(b)). The children underwent health examinations at the Seoul National University Hospital located in Jongno-gu, Seoul, Republic of Korea. The mothers’ depressive symptoms and children’s dietary habits were assessed using the Center for Epidemiologic Studies Depression (CES-D) [34] and food frequency questionnaires (FFQs) [35], respectively.

Neurobehavioral tests for social impairments and attention deficit hyperactivity disorder were administered in 2014 to the 4-year-old children. Therefore, social impairments were measured only in the 425 children who were followed up since 2014, but not in the 220 children who were followed up in 2013. Social impairments were assessed using the Korean version of the Social Communication Questionnaire (K-SCQ). However, 12 children did not complete this questionnaire; therefore, 413 children had valid social impairment scores at 4 years of age.

At the follow-up examination, urine and blood sample collections, as well as physical examinations, were conducted after the children had fasted for more than 8 h. After excluding those without maternal (*n* = 93) or child (*n* = 1) BPA measurements and those with gestational ages <36 weeks (*n* = 13) or unknown gestational ages (*n* = 2), 304 mother-child pairs were included in the present analyses. We obtained informed consent from all participants and the study protocol was approved by the Institutional Review Board at the College of Medicine, Seoul National University (IRB No. 1201-010-392).

### Social impairment

Symptoms of social impairment were measured using the validated K-SCQ [36, 37], which was completed by parents or caregivers. The K-SCQ has previously been used to screen for social impairments in children [38]. The K-SCQ consists of 40 items scored as “1” if the child has the item-specific social impairment symptom or “0” if that symptom is not present. The K-SCQ items can be divided into 3 subcategories of social impairments: social interaction, social communication, and behavior patterns (including restricted, repetitive, and stereotyped patterns of behavior [36]. Total scores and sub-scores for social impairment were calculated by summing the response scores for all applicable items. Higher total measure scores indicate a greater number of social impairment symptoms. Although total scores above a cutoff of 15 suggest that the individual is likely to have ASD [38], we could not investigate the relationship between ASD and BPA exposure because none of the children in the study had a score greater than 15.

### Exposure

Maternal spot urine samples were collected in conical tubes (SPL Lifesciences, Pocheon, Gyunggi-do, Republic of Korea) during the second trimester of pregnancy, between 14 and 27 weeks (mean of 20 weeks) of gestation. Children’s urine samples were collected after 8 h of fasting. The samples were sent to the laboratory (Seegene Medical Foundation, Seoul, Republic of Korea) and stored at −20 °C. We measured the total concentrations (free and conjugated species) of urinary BPA. Urine samples were treated with β-glucuronidase/sulfatase to hydrolyze conjugated BPA species [13]. BPA concentrations were quantified using high-performance liquid chromatography-tandem mass spectrometry (Agilent 6410 Triple Quad LCMS; Agilent, Santa Clara, CA, USA), as described previously [39, 40]. Standard solutions with BPA concentrations of 50, 25, 12.5, 6.25, 3.125, and 1.5625 μg/L were prepared and analyzed along with blanks to determine the standard calibration curve (r^2^ > 0.999). When the measured sample concentration was above the maximum concentration of the standard solution, the extract was diluted in water (1:1), separated into halves, and analyzed. One portion was subject to re-analysis if the detected concentration was not within 20% of the standard calibration curve. The lower limit of detection (LOD) for BPA ranged from 0.031 to 0.212 μg/L, depending on the batch used. We used an LOD of 0.212 μg/L divided by the square root of 2. We used creatinine-adjusted BPA concentrations in units of μg/g of creatinine in analyses to assess variations in urine concentrations of BPA [1]. Postnatal urinary BPA concentrations at the time of follow-up (approximately age 4) were measured using the same method used to measure maternal urinary BPA. Prenatal and postnatal BPA exposures were natural log-transformed for normality.

### Covariates

Potential covariates for inclusion in the statistical models were selected a priori, following a literature review [12, 13, 41]. Prenatal information was obtained using questionnaires at the time of recruitment; variables of interest included maternal age (years), gestational age (weeks), smoking (yes or no), drinking alcohol during pregnancy (yes or no), educational attainment (≤ or > than high school), parity (first vs. second or later child), and CES-D scores (0–60 points). Children’s characteristics such as age (months), gender, BMI (kg/m^2^), birth weight (kg), child-care (home, daycare, or other), exposure to second hand tobacco smoke (yes or no), and infant feeding type (breast feeding, bottle feeding, or mixed) were obtained at the follow-up visit. From various dietary habits queried by the FFQs, we selected those that were likely to be associated with BPA levels (*P*-value <0.1), including canned food or drinks (< or ≥1 per week), instant rice (< or ≥1 per week), and use of plastic dishes in the microwave oven (yes or no).

Covariates were first determined by searching for variables that reduced the Akaike information criterion (AIC) [42] in the model by >10%, compared to the base model (prenatal and postnatal BPAs were independent variables in the base model). Second, we selected variables that were significantly associated with total SCQ scores (*P*-value <0.05) after controlling for other covariates. In the final model, covariates included gender, parity, maternal education, birth weight, and use of plastic dishes in the microwave oven. We also controlled for prenatal and postnatal levels of urinary BPA.

### Statistical analyses

Generalized additive models (GAMs) were constructed to investigate the relationship between prenatal and postnatal BPA concentrations and social impairments at age 4. BPA has non-monotonic effects [41, 43]; therefore, we constructed 2 models, including 1 with a linear BPA term and another spline model for BPA exposure (4 degrees of freedom). When the shape of the association in GAMs looked nonlinear, we compared the AIC of linear and spline models to select a better-fit model for a given set of data. We also calculated the difference of deviances for the fitted models and tested statistical significance of the difference, which followed a chi-square distribution.

After visualizing the relationship between BPA exposure and social impairments, we estimated the contributions of BPA to the linear and piecewise linear regression models. First, to estimate the overall linear effects of BPA on social impairments, we constructed regression models for BPA exposure and social impairments. Second, we estimated prenatal and postnatal BPA effects at concentrations that were either less than, or equal to/greater than, threshold BPA concentrations, using piecewise linear regression models and the *threshpt* function in the *HEAT* package [44] of R software (R Development Core Team, https://cran.r-project.org/). Piecewise linear regression analysis has been used to determine flexion points in non-linear relationships [41, 45, 46] using AIC as a measure of the relative quality of a statistical model for a given set of data. We modeled scores on the K-SCQ as a Poisson distribution and estimated the effects of BPA on the total and subcategory scores for social impairments (social interaction, social communication, and behavior patterns). All models were controlled for gender, parity, maternal education, birth weight, use of plastic dishes in the microwave oven, and prenatal or postnatal levels of urinary BPA. To examine gender differences in our analyses, we stratified our samples by children’s gender.

In our sensitivity analysis, we examined the association between prenatal and postnatal concentrations of BPA and children’s social impairments at age 4, using BPA concentrations (μg/L) unadjusted for creatinine, and using the same covariates as the main analyses. Instead of directly adjusting for BPA concentrations, the urinary creatinine levels of the mothers and children were included in the model. Finally, we compared percentage changes in SCQ total scores associated with prenatal and postnatal BPA, with or without adjusting for covariates that were excluded in the final model; the covariates included maternal age, gestational age, smoking during pregnancy, drinking alcohol during pregnancy, mother’s depression, child’s age, infant feeding type, second hand smoke, place of childcare, canned food or drink, and instant rice. All analyses were conducted using SAS (v9.4; Cary, NC, USA) and the R software package (v3.2.1). Two-tailed *P* values <0.05 were considered statistically significant.

## Results

Characteristics of mothers (*n* = 615) included in the present EDC study were different from excluded mothers (*n* = 10,137) in the CAS cohort; differences in the EDC cohort included that the mothers were older (31.2 years vs. 30.6 years for included and excluded mothers, respectively), children were born at an earlier gestational age (39.2 weeks vs. 39.3 weeks), there were more twin or triplets (3.9% vs. 1.7%), and there were more current or past smokers (45.6 vs. 41.3) (Additional file 1: Table S1). Characteristics including maternal age, prenatal BPA levels, and K-SCQ scores of the children in the present study (*N* = 304) were similar to those of the excluded children (*N* = 341). However, the included children were slightly younger (47.7 vs. 48.0 months; *P* = 0.0270) and had lower creatinine-adjusted BPA levels at 4 years of age (4.9 vs. 5.7 μg/g creatinine; *P* = 0.0006) compared to the excluded children (Additional file 1: Table S1).

The mean age of the 304 mothers was 31.2 years, and 82.9% of the mothers had higher than a high school education. The children included 52.6% boys, 63.8% of which were the first child in the family. Only 52.1% of the girls were the first child in the family. The mean score for depressive symptoms was 11.4. The mean creatinine-adjusted BPA exposure at the midterm of the pregnancy was 2.0 μg/g creatinine (Table 1). Approximately one-quarter of the children were exposed to second hand tobacco smoke. Among dietary habits, only canned food or drink consumption was higher in boys than in girls. The mean creatinine-adjusted BPA level in the 4-year-old children was 4.9 μg/g creatinine. A greater total number of social impairment symptoms were observed in the boys compared to girls. This gender difference was statistically significant (5.0 vs. 3.6, respectively; *P* = 0.0001). The difference was predominantly attributed to the behavior patterns subcategory (1.7 vs. 1.1 in boys and girls, respectively) (Table 2). Creatinine adjusted prenatal and postnatal BPA concentrations were not significantly correlated (Pearson’s correlation = 0.01136).

**Table 1:** Characteristics of participants’ mothers (*N* = 304)

Variables	Mean ± SD, N (%)			*P*-value
	Overall	Boys (*n* = 160)	Girls (*n* = 144)	
Maternal information				
Mother’s age (years)	31.2 ± 3.6	31.4 ± 3.7	31.0 ± 3.4	0.3873
Gestational age (weeks)	39.2 ± 1.2	39.2 ± 1.2	39.2 ± 1.2	0.9349
Smoking during pregnancy	7 (2.4)	4 (2.6)	3 (2.2)	1.0000
Drinking alcohol during pregnancy	28 (9.7)	21 (13.6)	7 (5.2)	0.0172
Mother’s education				0.4490
> High school	252 (82.9)	130 (81.3)	122 (84.7)	
≤ High school	52 (17.1)	30 (18.8)	22 (15.3)	
Parity				<.0001
First child	177 (58.2)	102 (63.8)	75 (52.1)	
Second +	127 (41.8)	58 (36.3)	69 (47.9)	
Depression score (0–60)	11.4 ± 7.9	11.2 ± 8.1	11.5 ± 7.6	0.8108
Creatinine (μg/L)	87.9 ± 50.3	88.0 ± 49.2	87.7 ± 51.7	0.9531
Creatinine-adjusted prenatal BPA (μg/g creatinine)	2.0 ± 2.1	2.3 ± 2.3	1.6 ± 1.7	0.0088

*Abbreviations*: *SD* standard deviation, *BPA* bisphenol A

**Table 2:** Characteristics of children in the present study (*N* = 304)

Variables	Mean ± SD, N (%)			*P*-value
	Overall	Boys (*n* = 160)	Girls (*n* = 144)	
Child’s age (months)	47.7 ± 2.1	47.7 ± 2.0	47.8 ± 2.1	0.4569
Body mass index	15.7 ± 1.3	15.8 ± 1.2	15.7 ± 1.3	0.4508
Birth weight (kg)	3.3 ± 0.4	3.3 ± 0.4	3.2 ± 0.4	0.0274
Feeding				0.8972
Breast feeding only	77 (34.5)	38 (31.7)	39 (37.9)	
Breast and bottle feeding	124 (55.6)	73 (60.8)	51 (49.5)	
Bottle feeding only	22 (9.9)	9 (7.5)	13 (12.6)	
Place of childcare				0.5199
Home	177 (65.8)	94 (64.0)	83 (68.0)	
Daycare or other	92 (34.2)	53 (36.1)	39 (32.0)	
Second hand smoke exposure				0.0477
No	221 (72.7)	115 (71.9)	106 (73.6)	
Yes	83 (27.3)	45 (28.1)	38 (26.4)	
Canned food or drink				0.0011
< 1 per week	259 (85.2)	126 (78.8)	133 (92.4)	
≥ 1 per week	45 (14.8)	34 (21.3)	11 (7.6)	
Instant rice				0.1246
< 1 per week	300 (98.7)	156 (97.5)	144 (100.0)	
≥ 1 per week	4 (1.3)	4 (2.5)		
Use of plastic dishes in the microwave oven				0.8831
No	247 (81.3)	129 (80.6)	118 (81.9)	
Yes	57 (18.8)	31 (19.4)	26 (18.1)	
BPA at 4 years of age (μg/L)	3.2 ± 5.0	3.2 ± 3.1	3.3 ± 6.6	0.8144
Creatinine (μg/L)	72.1 ± 34.9	74.9 ± 35.3	69.1 ± 34.3	0.1483
Creatinine-adjusted BPA at 4 years of age (μg/g creatinine)	4.9 ± 10.8	4.3 ± 3.6	5.5 ± 15.3	0.3366
K-SCQ- Total	4.3 ± 3.1	5.0 ± 3.2	3.6 ± 2.7	0.0001
K-SCQ- Social interaction	1.1 ± 1.2	1.2 ± 1.3	0.9 ± 1.0	0.0083
K-SCQ- Communication	1.7 ± 1.4	1.8 ± 1.4	1.6 ± 1.3	0.0956
K-SCQ- Behavior Patterns	1.4 ± 1.7	1.7 ± 1.7	1.1 ± 1.5	0.0014

*Abbreviations*: *SD* standard deviation, *BPA* bisphenol A, *K-SCQ* Korean version of the Social Communication Questionnaire

**Fig. 1:**
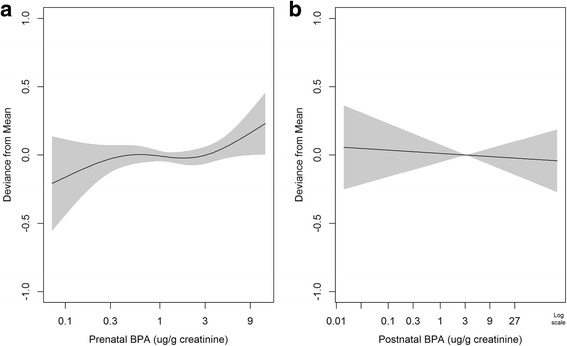
Relationship between creatinine-adjusted concentrations of prenatal (**a**) and postnatal (**b**) bisphenol A (BPA) and total Korean version of the Social Communication Questionnaire (K-SCQ) scores. Models were adjusted for gender (boy or girl), parity (1st vs. other), mother’s education (high school or lower vs. more than high school), birth weight (kg), and use of plastic dishes in the microwave oven (yes or no). In addition, prenatal and postnatal BPA levels were mutually controlled for in the model

Figure 1(a) and (b) show the associations between prenatal and postnatal BPA and K-SCQ scores, respectively. The regression curve deviates from a straight line and has a flexion point at around 3.0 μg/g creatinine of prenatal BPA at the mid-term of pregnancy (Fig. 1(a)). AIC in the spline model with 4 degrees of freedom was slightly smaller compared to the linear model (1495 for the spline model vs. 1497 for the linear model), suggesting that the spline model is a better fit to assess prenatal BPA and social impairments. The difference in deviance was statistically significant (*P* = 0.0284). Furthermore, 18% of mothers (55 of 304) had values above this level of 3.0 μg/g creatinine, which is 1.5 times greater than the mean prenatal BPA concentration in our sample. The maximum BPA level was 13.0 μg/g creatinine. The assumption of linearity was met for the association between social impairments and postnatal BPA levels at 4 years of age (Fig. 1(b)). AIC values were 1498 for the spline model with 4 degrees of freedom and 1495 for the linear model, suggesting that the linear model is a better fit for our data regarding the association between postnatal BPA and social impairment. Difference of deviance was not statistically significant (*P* = 0.2391).

Table 3 presents estimations of linear relationships between prenatal and postnatal BPA exposure and the total and subcategory scores for social impairments. We did not observe significant linear associations between prenatal BPA exposure and social impairments, as previously shown in Fig. 1(a). However, each doubling of postnatal exposure to BPA at 4 years of age was significantly associated with an increase in social communication of 11.8% (95% confidence interval [CI], 0.6% to 24.3%) in girls. The effect size was significantly different in girls compared to boys (*P* = 0.0054). Our sensitivity analysis indicated that there is a similar relationship for creatinine-unadjusted postnatal BPA as for creatinine-adjusted BPA (Additional file 1: Table S3). Additional adjustment for covariates did not change the main findings (Additional file 1: Table S4).

**Table 3:** Percentage change in scores on the K-SCQ associated with 2-fold increases in creatinine-adjusted prenatal and postnatal BPA levels

Subcategory of K-SCQ score	Gender	Prenatal BPA			Postnatal BPA		
		% change (95% CI)	*P*-value	*P*-value for gender difference	% change (95% CI)	*P*-value	*P*-value for gender difference
Total	All	3.4 (−0.8, 7.7)	0.1134	0.2464	−1.0 (−5.1, 3.3)	0.6517	0.0034
	Boys	3.7 (−1.4, 9.1)	0.1593		−5.2 (−10.3, 0.1)^#^	0.0553	
	Girls	2.2 (−4.6, 9.4)	0.5359		4.5 (−2.3, 11.8)	0.1966	
Social interaction	All	−0.3 (−8.1, 8.1)	0.9365	0.3835	−5.6 (−13.1, 2.5)	0.1708	0.8667
	Boys	−3.2 (−12.1, 6.7)	0.5129		−6.8 (−16.4, 3.8)	0.2003	
	Girls	7.0 (−7.0, 23.2)	0.3434		−5.7 (−17.4, 7.8)	0.3904	
Social communication	All	3.5 (−3.1, 10.6)	0.3090	0.2167	2.0 (−4.9, 9.3)	0.5848	0.0054
	Boys	6.4 (−2.3, 15.8)	0.1548		−5.2 (−13.5, 3.9)	0.2551	
	Girls	−1.0 (−10.8, 9.9)	0.8526		11.8 (0.6, 24.3)^*^	0.0392	
Behavior patterns	All	5.5 (−1.9, 13.5)	0.1463	0.2075	−0.2 (−7.5, 7.6)	0.9510	0.0374
	Boys	6.0 (−3.1, 16.1)	0.2020		−4.8 (−13.4, 4.5)	0.2999	
	Girls	3.2 (−8.7, 16.7)	0.6146		5.2 (−6.7, 18.6)	0.4102	

Models were adjusted for gender (boy or girl), parity (1st vs. other), mother’s education (high school or lower vs. more than high school), birth weight (kg), and use of plastic dishes in the microwave oven (yes or no). In addition, prenatal and postnatal BPA levels were mutually controlled for in the model

*Abbreviations*: *K-SCQ* Korean version of the Social Communication Questionnaire, *BPA* bisphenol A, *CI* confidence interval

^*^ *P*-value <0.05; ^#^ *P*-value <0.1

We observed a flexion point in the association curve in Fig. 1(a); therefore, we estimated the above-threshold effects of prenatal BPA on social impairments. At or above the threshold (3.0 μg/g creatinine of BPA), the total scores of all participants increased by 16.9% (95% CI, 2.3% to 33.5%) per 2-fold increase in prenatal BPA. The magnitude of the increase in social communication scores associated with prenatal BPA concentrations at or above the threshold (3.0 μg/g creatinine of BPA) was greater in girls than boys (4.7% [95% CI, −22.4% to 41.3%] for boys vs. 58.4% [95% CI, 6.5% to 135.8%] for girls) (Table 4). This difference was marginally significant (*P*-value <0.1) (Additional file 1: Figure S2). Social interaction and behavior patterns were not associated with prenatal or postnatal BPA concentrations in the total sample or for boys and girls separately.

**Table 4:** Percentage change in K-SCQ scores associated with 2-fold increases in creatinine-adjusted prenatal BPA levels above or below 3.0 μg/g creatinine

		Below 3.0 μg/g creatinine		At or above 3.0 μg/g creatinine	
Subcategory of K-SCQ score	Gender	% change (95% CI)	*P*-value	% change (95% CI)	*P*-value
Total	All	−0.1 (−4.8, 4.9)	0.9728	16.9 (2.3, 33.5)^*^	0.0214
	Boys	1.3 (−5.2, 8.2)	0.7062	10.8 (−6.1, 30.7)	0.2248
	Girls	−1.7 (−8.8, 6.0)	0.6624	29.4 (0.2, 67.1)^*^	0.0483
Social interaction	All	−1.6 (−11.6, 9.6)	0.7707	11.4 (−17.5, 50.4)	0.4817
	Boys	−6.4 (−18.2, 7.1)	0.3341	15.8 (−18.5, 64.7)	0.4127
	Girls	13.2 (−5.7, 35.8)	0.1842	−3.4 (−51.6, 92.5)	0.9210
Social communication	All	−1.3 (−9.5, 7.6)	0.7638	24.4 (−1.0, 56.4)^#^	0.0610
	Boys	7.0 (−5.6, 21.3)	0.2886	4.7 (−22.4, 41.3)	0.7644
	Girls	−9.0 (−20.0, 3.5)	0.1516	58.4 (6.5, 135.8)^*^	0.0233
Behavior patterns	All	2.2 (−7.3, 12.7)	0.6554	20.2 (−7.4, 55.9)	0.1667
	Boys	3.7 (−8.9, 18.1)	0.5796	16.3 (−15.0, 59.2)	0.3443
	Girls	−0.9 (−15.1, 15.7)	0.9080	29.3 (−24.5, 121.6)	0.3488

Models were adjusted for gender (boy or girl), parity (1st vs. other), mother’s education (high school or lower vs. more than high school), birth weight (kg), and use of plastic dishes in the microwave oven (yes or no). In addition, prenatal and postnatal BPA levels were mutually controlled for in the model

*Abbreviations*: *K-SCQ* Korean version of the Social Communication Questionnaire, *BPA* bisphenol A, *CI* confidence interval

^*^ *P*-value <0.05; ^#^ *P*-value <0.1

## Discussion

The present study examined the relationships between prenatal and postnatal BPA exposure, and social impairments in 4-year-old children; we found that prenatal exposure to BPA at or above 3.0 μg/g creatinine and postnatal exposure to BPA at 4 years of age were significantly associated with social communication impairments in girls, as measured by the K-SCQ.

Exposure to endocrine-disrupting chemicals with estrogenic activity can alter brain and behavioral development during critical periods of fetal development [47]. Rodent studies have demonstrated that prenatal exposure to BPA affects social behaviors, anxiety levels, and sexual differentiation in offspring [48, 49] through enduring transgenerational effects on vasopressin and oxytocin mRNA in the brain [50]. These hormones affect many social behaviors [51, 52]. However, human studies have produced inconsistent results. Two studies have shown that prenatal exposure to BPA is associated with depressed behavior and anxiety in 3-year-old children [12], as well as internalizing and externalizing problems in school-aged children [16]. However, other studies have not found significant associations between prenatal exposure to BPA and social impairments [19] or autistic behaviors [20] in school-aged children.

While toxicology studies frequently assume the presence of linear relationships, this assumption may not be valid for receptor-mediated mechanisms [53]. We found that the linear association between social impairment and prenatal BPA was not statistically significant at the α = 0.05 level. However, considering the flexion point in the non-linear relationship, total K-SCQ was strongly associated with above-threshold prenatal BPA concentrations (16.9% [95% CI, 2.3% to 33.5%]). This association was particularly strong for social communication in girls (58.4% [95% CI, 6.5% to 135.8%]). The range of BPA exposures in this study was quite narrow compared to the doses typically employed in animal neurotoxicity studies [53, 54]. Therefore, we cannot conclude that the non-linear relationship indicated by our study is consistent with non-linear relationships in animal neurotoxicity studies. However, the non-linear relationship is similar to the findings of other human observational studies with low-level exposure to BPA, which are comparable to the exposure level in the present study [25, 41]. To confirm the threshold effects, further studies of the non-linear relationships between neurobehavioral development and exposure to BPA should be conducted during the critical period, including in other geographic areas or in populations of other races or ethnicities.

One previous study has evaluated the association between social impairments or autistic behavior and childhood exposure to BPA. The results of that study indicate that BPA metabolites are 2 times higher in children with ASD compared to children without ASD [55]. Our results indicate that there is a significant association between postnatal exposure to BPA and social communication, but not between BPA and other subcategories of social impairments (social interaction and behavior patterns). However, the scarcity of prior studies examining the link between BPA exposure in children and the risk of social impairments or autistic behavior, combined with the fact that the current study found a significant association only for a subtype of social impairment, necessitate additional epidemiological studies to confirm our findings.

Prenatal BPA exposure alters mRNA for the epigenetic regulators DNA methyltransferase 1 (DNMT1) and DNMT3A, as well as the brain region-specific expression of genes encoding estrogen receptors. Therefore, BPA may underlie enduring changes in brain function and behavior, especially for sexually dimorphic phenotypes [56]. However, epidemiological studies of sex-specific BPA effects on behavior have produced inconsistent results. Two previous studies found significant associations between prenatal BPA exposure and behavioral changes only in girls [12, 13]. Conversely, other studies have found significant associations between prenatal BPA and anxiety, depression, and behavioral changes in boys, but not girls [14–16, 57]. When using non-linear regression models, we observed significantly greater effects of prenatal BPA at or above the threshold level (3.0 μg/g creatinine) on social communication in girls compared to boys (58.4% for girls vs. 4.7% for boys). Similarly, we observed a significant gender difference in the association between postnatal exposure to BPA and social communication. Further studies may be required to address the differential mechanisms for the effects of exposure to BPA on neurobehavioral development in boys compared to girls.

Although the cohort design of this study is a strength, our study also had some limitations. First, the K-SCQ was used as a screening tool to detect social impairments in children. As this test was completed by parents or caregivers, its results may have been influenced by observer bias. Furthermore, none of the children in the current study had severe social impairments at 4 years of age. Parents or caregivers may over- or under-report their children’s symptoms, which may result in misclassification on the K-SCQ and null associations. Given this limitation, the present study conservatively estimated the effects of prenatal and postnatal BPA exposure and found significant associations between BPA exposure and social impairment at 4 years of age. Second, we collected spot urine samples from pregnant women and their children. Given that the half-life of BPA is approximately 6 h, the spot urine samples may not have captured intra-individual variability in BPA metabolism over time; however, Ye et al. propose that spot urine samples reflect the average exposure of a population to BPA [58]. We collected urine samples in the morning to eliminate within-day variations, although unmeasured variation may still exist. Third, since social skills in children may progress as they develop, caution should be exercised in the interpretation of the results obtained at 4 years of age, as social communication skills have not matured. Finally, we did not investigate the potential for reverse causality of the association between postnatal BPA and social impairments. Children with more behavior problems may have different dietary or mouthing behavior [59, 60] that may increase their BPA exposure.

## Conclusions

The prospective cohort study design is a strength of this study investigating the relationships between prenatal and postnatal BPA concentrations and social impairments at 4 years of age. Although the study has several limitations, including parent-reported questionnaires to evaluate social impairments and no participants with severe social impairments, the study makes a significant contribution to research on endocrine disruptors’ impact on children health because the relationship between BPA exposure and neurodevelopmental effects has not been fully elucidated in humans, and our results elucidate BPA exposure effects related to social impairments. Specifically, prenatal BPA exposure was significantly associated with impairments at or above the flexion point of 3.0 μg/g creatinine, whereas there was a linear association for postnatal BPA exposure. Further studies to evaluate the health implications and underlying mechanisms of these findings are warranted.

## Additional file

Additional file 1: Figure S1. Selection of study participants. **Table S1.** Characteristics of mothers included and excluded in the follow-up of the Birth cohort (*N* = 10,752). **Table S2.** Characteristics of participants included and excluded in the study. **Table S3.** Percentage change in scores on the K-SCQ associated with 2-fold increase in creatinine-unadjusted prenatal and postnatal BPA levels. **Table S4.** Percentage change in scores on the K-SCQ associated with 2-fold increase in creatinine-adjusted prenatal and postnatal BPA levels after controlling for covariates. **Figure S2.** Percentage change in scores on the K-SCQ associated with 2-fold increase at or above level of creatinine-adjusted prenatal Bisphenol A (3.0 μg/g creatinine) by sex. (DOCX 143 kb)

## Abbreviations

ASD: Autism spectrum disorder
BMI: Body mass index
BPA: Bisphenol A
DNMT: DNA methyltransferase
EDC: Environment and Development of Children
K-SCQ: Korean version of the Social Communication Questionnaire
LOD: Lower limit of detection
