# Supplementary material for: Prenatal and postnatal bisphenol A exposure and social impairment in 4-year-old children
Source: Environ Health. 2017 Jul 26;16:79. doi: 10.1186/s12940-017-0289-2 (PMC5530578; doi:10.1186/s12940-017-0289-2)
Supplement: Additional file 1: Figure S1. — Selection of study participants. Table S1. Characteristics of mothers included and excluded in the follow-up of the Birth cohort (N = 10,752). Table S2. Characteristics of participants included and excluded in the study. Table S3. Percentage change in scores on the K-SCQ associated with 2-fold increase in creatinine-unadjusted prenatal and postnatal BPA levels. Table S4. Percentage change in scores on the K-SCQ associated with 2-fold increase in creatinine-adjusted prenatal and postnatal BPA levels after controlling for covariates. Figure S2. Percentage change in scores on the K-SCQ associated with 2-fold increase at or above level of creatinine-adjusted prenatal Bisphenol A (3.0 μg/g creatinine) by sex. (DOCX 143 kb) [file 12940_2017_289_MOESM1_ESM.docx]

**Additional file 1**

**Prenatal and postnatal bisphenol A exposure and social impairment in 4-year-old children**

Youn-Hee Lim^1,2^, Sanghyuk Bae^3^, Bung-Nyun Kim^4^, Choong Ho Shin^5^, Young Ah Lee^5^, Johanna Inhyang Kim^4^, Yun-Chul Hong^1,2,6,^*

^1^Institute of Environmental Medicine, Seoul National University Medical Research Center, Seoul, Republic of Korea

^2^Environmental Health Center, Seoul National University College of Medicine, Seoul, Republic of Korea

^3^Department of Preventive Medicine, Dankook University, Cheonan, Republic of Korea

^4^Division of children and Adolescent Psychiatry, Department of Psychiatry, Seoul National University Hospital, Seoul, Republic of Korea

^5^Department of Pediatrics, Seoul National University Children’s Hospital, Seoul, Republic of Korea

^6^Department of Preventive Medicine, Seoul National University College of Medicine, Seoul, Republic of Korea

**^*^**Address correspondence to Yun-Chul Hong, Institute of Environmental Medicine, Seoul National University Medical Research Center, 103 Daehakro, Jongno-Gu, Seoul 110-799, Republic of Korea

Telephone: +82-2-740-8394; Fax: +82-2-747-4830; E-mail: [ychong1@snu.ac.kr](mailto:ychong1@snu.ac.kr)

**Table of Contents**

**Tables & Figures**

[Figure S1. Selection of study participants 3](#_Toc486596826)

[Table S1. Characteristics of mothers included and excluded in the follow-up of the Birth cohort (N=10,752) 4](#_Toc486596827)

[Table S2. Characteristics of participants included and excluded in the study 5](#_Toc486596828)

[Table S3. Percentage change in scores on the K-SCQ associated with 2-fold increase in creatinine-unadjusted prenatal and postnatal BPA levels 7](#_Toc486596829)

[Table S4. Percentage change in scores on the K-SCQ associated with 2-fold increase in creatinine-adjusted prenatal and postnatal BPA levels after controlling for covariates 8](#_Toc486596830)

[Figure S2. Percentage change in scores on the K-SCQ associated with 2-fold increase at or above level of creatinine-adjusted prenatal Bisphenol A (3.0 μg/g creatinine) by sex 9](#_Toc486596831)

Figure S1. Selection of study participants

1. Flow chart of selection


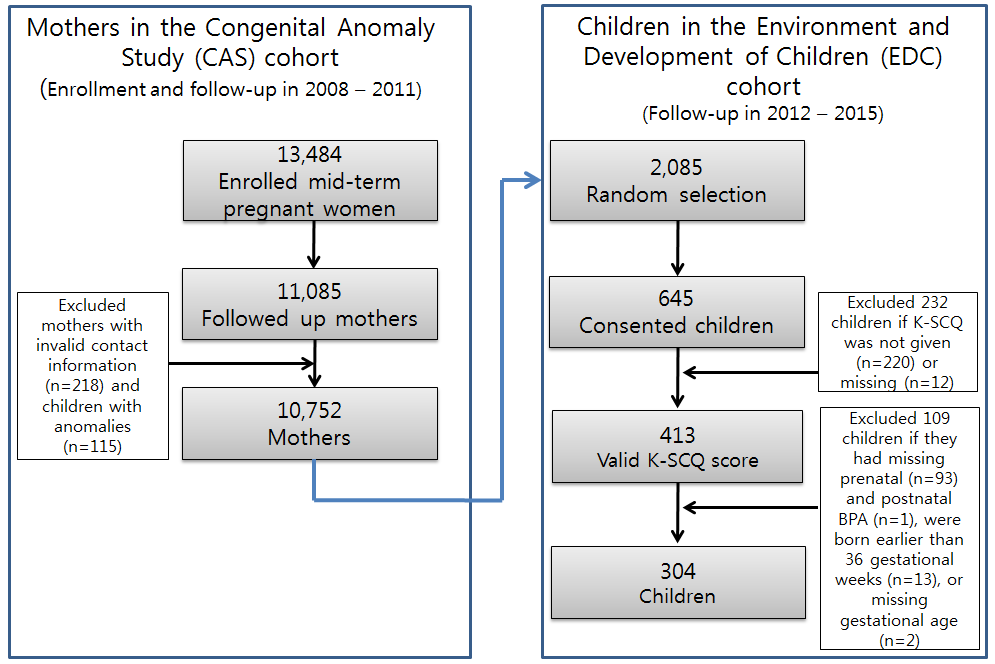


(b) Number of followed-up children in the EDC cohort by birth year


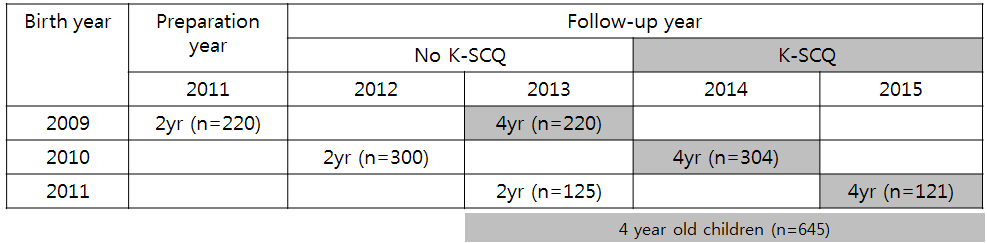


Table S1. Characteristics of mothers included and excluded in the follow-up of the Birth cohort (N=10,752)

| Variables | Mean ± SD, n(%) | | | P-value |
| --- | --- | --- | --- | --- |
|  | Overall (n=10,752) | Inclusion (n=615) | Exclusion (n=10,137) |  |
| Mother age (Years) | 30.6 ± 3.5 | 31.2 ± 3.6 | 30.6 ± 3.5 | <.0001 |
| Gestational age (Weeks) | 39.3 ± 3.2 | 39.2 ± 1.4 | 39.3 ± 3.3 | 0.0227 |
| Twin+ | 191 (1.8) | 24 (3.9) | 167 (1.7) | <.0001 |
| Smoking during pregnancy | 589 (5.6) | 7 (1.2) | 284 (2.8) | 0.0175 |
| Drinking alcohol during pregnancy | 2283 (21.9) | 28 (1.2) | 262 (3.2) | <.0001 |

Table S2. Characteristics of participants included and excluded in the study

| Variables | Mean ± SD,  n (%) | | | P-value |
| --- | --- | --- | --- | --- |
|  | Overall (n=645) | Inclusion (n=304) | Exclusion (n=341) |  |
| ***Maternal information*** |  |  |  |  |
| Mother age (Years) | 31.2 ± 3.6 | 31.2 ± 3.6 | 31.3 ± 3.6 | 0.8359 |
| Gestational age (Weeks) | 39.0 ± 1.6 | 39.23 ± 1.19 | 38.85 ± 1.80 | 0.0019 |
| Smoking at pregnancy | 590 (5.6) | 7 (2.4) | 583 (5.6) | 0.0132 |
| Alcohol drink at pregnancy | 2285 (21.9) | 28 (9.7) | 2257 (22.2) | <.0001 |
| Mother's education |  |  |  | 0.5404 |
| > high school | 528 (81.9) | 252 (82.9) | 276 (80.9) |  |
| ≤ high school | 117 (18.1) | 52 (17.1) | 65 (19.1) |  |
| Prenatal BPA (μg/L) | 1.7 ± 2.6 | 1.7 ± 2.4 | 1.8 ± 2.9 | 0.7268 |
| Maternal creatinine (μg/L) | 86.9 ± 50.6 | 87.9 ± 50.3 | 85.6 ± 50.9 | 0.6090 |
| Creatinine-adjusted prenatal BPA  (μg/g creatinine) | 2.0 ± 2.6 | 2.0 ± 2.1 | 2.1 ± 3.1 | 0.6993 |
|  |  |  |  |  |
| ***Children’s information*** |  |  |  |  |
| Child age (Months) | 47.9 ± 1.8 | 47.7 ± 2.1 | 48.0 ± 1.4 | 0.0270 |
| Sex |  |  |  |  |
| Boy | 338 (52.4) | 160 (52.6) |  |  |
| Girl | 307 (47.6) |  | 163 (47.8) |  |
| Body mass index (kg/m^2^) | 15.6 ± 1.3 | 15.7 ± 1.3 | 15.5 ± 1.3 | 0.0230 |
| Parity |  |  |  | 0.9371 |
| First child | 366 (56.8) | 177 (58.2) | 189 (55.6) |  |
| Second + | 278 (43.2) | 127 (41.8) | 151 (44.4) |  |
| Secondhand smoking |  |  |  | 0.5243 |
| No | 484 (75.0) | 221 (72.7) | 263 (77.1) |  |
| Yes | 161 (25.0) | 83 (27.3) | 78 (22.9) |  |
| BPA at age 4 years (μg/L) | 3.8 ± 5.7 | 3.2 ± 5.0 | 4.3 ± 6.2 | 0.0183 |
| Creatinine (μg/L) | 73.0 ± 35.1 | 72.1 ± 34.9 | 73.8 ± 35.3 | 0.5383 |
| Creatinine-adjusted BPA at age 4 years (μg/g creatinine) | 5.0 ± 5.1 | 4.9 ± 10.8 | 5.7 ± 5.5 | 0.0006 |
| K-SCQ- Total | 4.4 ± 3.1 | 4.3 ± 3.1 | 4.4 ± 3.2 | 0.9607 |
| K-SCQ- Social interaction | 1.1 ± 1.2 | 1.1 ± 1.2 | 1.2 ± 1.3 | 0.2859 |
| K-SCQ- Communication | 1.7 ± 1.4 | 1.7 ± 1.4 | 1.8 ± 1.5 | 0.6769 |
| K-SCQ- Social interaction | 1.4 ± 1.6 | 1.38 ± 1.68 | 1.48 ± 1.42 | 0.5686 |

Abbreviations: SD, standard deviation; BPA, Bisphenol A; K-SCQ, Korean version of the Social Communication Questionnaire

Table S3. Percentage change in scores on the K-SCQ associated with 2-fold increase in creatinine-unadjusted prenatal and postnatal BPA levels

|  | Subcategory of SCQ score | Prenatal BPA | | | Postnatal PBA | | |
| --- | --- | --- | --- | --- | --- | --- | --- |
|  |  | % change (95% CI) | P-value | P-value for sexual difference | % change (95% CI) | P-value | P-value for sexual difference |
| Total | Overall | 3.7 (-0.5, 8.1)# | 0.0814 | 0.1397 | -1.1 (-5.2, 3.3) | 0.6222 | 0.0911 |
|  | Male | 4.6 (-0.7, 10.1)# | 0.0915 |  | -6.0 (-11.1, -0.7)* | 0.0283 |  |
|  | Female | 2.4 (-4.4, 9.7) | 0.4914 |  | 4.6 (-2.2, 11.9) | 0.1910 |  |
| Social interaction | Overall | -0.2 (-8.0, 8.4) | 0.9701 | 0.3776 | -5.6 (-13.1, 2.5) | 0.1693 | 0.7291 |
|  | Male | -2.0 (-11.2, 8.1) | 0.6864 |  | -7.5 (-17.0, 3.0) | 0.1570 |  |
|  | Female | 6.0 (-8.0, 22.2) | 0.4209 |  | -7.0 (-18.8, 6.6) | 0.2966 |  |
| Social communication | Overall | 3.4 (-3.3, 10.6) | 0.3234 | 0.3899 | 2.0 (-4.9, 9.4) | 0.5760 | 0.2657 |
|  | Male | 6.4 (-2.5, 16.0) | 0.1654 |  | -5.3 (-13.7, 3.8) | 0.2440 |  |
|  | Female | -0.3 (-10.3, 10.9) | 0.9620 |  | 11.8 (0.5, 24.4)* | 0.0396 |  |
| Behavior | Overall | 6.7 (-0.8, 14.8)# | 0.0819 | 0.3620 | -0.8 (-8.1, 7.0) | 0.8289 | 0.0504 |
|  | Male | 7.8 (-1.5, 18.1) | 0.1034 |  | -6.7 (-15.2, 2.6) | 0.1507 |  |
|  | Female | 3.1 (-8.9, 16.7) | 0.6286 |  | 6.0 (-6.1, 19.6) | 0.3455 |  |

Abbreviations: K-SCQ, Korean version of the Social Communication Questionnaire; BPA, Bisphenol A; CI, confidence interval

Models were adjusted for gender (boy or girl), parity (1st vs. other), mother’s education (high school or lower vs. more than high school), birth weight (kg), use of plastic dishes in the microwave oven (yes or no), and maternal and postnatal creatinine. In addition, creatinine-unadjusted prenatal and postnatal BPA levels were mutually controlled for in the model.

* P-value < 0.05; # P-value <0.1

Table S4. Percentage change in scores on the K-SCQ associated with 2-fold increase in creatinine-adjusted prenatal and postnatal BPA levels after controlling for covariates

| Covariates | Sample size (n) | % change (95% CI) | | | |
| --- | --- | --- | --- | --- | --- |
|  |  | Prenatal BPA | | Postnatal BPA | |
|  |  | Model 1 | Model 2 | Model 1 | Model 2 |
| Maternal age | 304 | 3.4 (-0.8, 7.7) | 3.4 (-0.8, 7.7) | -1.0 (-5.1, 3.3) | -0.8 (-5.0, 3.5) |
| Gestational age | 304 | 3.4 (-0.8, 7.7) | 3.4 (-0.7, 7.8) | -1.0 (-5.1, 3.3) | -1.2 (-5.4, 3.1) |
| Smoking during pregnancy | 279 | 2.9 (-1.3, 7.3) | 2.1 (-2.2, 6.7) | -2.4 (-6.8, 2.2) | -3.4 (-7.8, 1.3) |
| Drinking alcohol during pregnancy | 290 | 2.9 (-1.3, 7.3) | 2.8 (-1.5, 7.2) | -2.4 (-6.8, 2.2) | -2.7 (-7.0, 1.9) |
| Mother's depression | 242 | 6.8 (2.0, 11.9) | 6.9 (2.0, 12.0) | -2.5 (-6.9, 2.2) | -2.7 (-7.2, 2.0) |
| Child's age | 304 | 3.4 (-0.8, 7.7) | 3.4 (-0.8, 7.8) | -1.0 (-5.1, 3.3) | -1.2 (-5.4, 3.1) |
| Infant feeding type | 223 | 8.6 (3.5, 13.9) | 8.8 (3.7, 14.3) | -2.7 (-7.0, 1.8) | -2.9 (-7.3, 1.6) |
| Body mass index | 304 | 3.4 (-0.8, 7.7) | 3.4 (-0.8, 7.7) | -1.0 (-5.1, 3.3) | -1.0 (-5.1, 3.3) |
| Secondhand Smoke | 304 | 3.4 (-0.8, 7.7) | 3.3 (-0.9, 7.6) | -1.0 (-5.1, 3.3) | -1.0 (-5.2, 3.3) |
| Place of childcare | 269 | 4.0 (-0.5, 8.6) | 4.0 (-0.5, 8.6) | -0.6 (-4.9, 3.9) | -0.6 (-5.0, 3.9) |
| Canned food or drinks | 304 | 3.4 (-0.8, 7.7) | 3.3 (-0.8, 7.7) | -1.0 (-5.1, 3.3) | -1.0 (-5.1, 3.3) |
| Instant rice | 304 | 3.4 (-0.8, 7.7) | 3.1 (-1.1, 7.4) | -1.0 (-5.1, 3.3) | -1.0 (-5.2, 3.3) |

Abbreviations: K-SCQ, Korean version of the Social Communication Questionnaire; BPA, Bisphenol A; CI, confidence interval

Model 1 was adjusted for gender (boy or girl), parity (1st vs. other), mother’s education (high school or lower vs. more than high school), birth weight (kg), and use of plastic dishes in the microwave oven (yes or no). In addition, creatinine-adjusted maternal and postnatal BPAs were mutually controlled for in the model.

Model 2 was additionally adjusted for one of the potential covariates (maternal age, gestational age, smoking and drinking alcohol during pregnancy, mother’s depression, child age, infant feeding type, body mass index, secondhand smoke, place of childcare, canned food or drinks, and instant rice).

Figure S2. Percentage change in scores on the K-SCQ associated with 2-fold increase at or above level of creatinine-adjusted prenatal Bisphenol A (3.0 μg/g creatinine) by sex


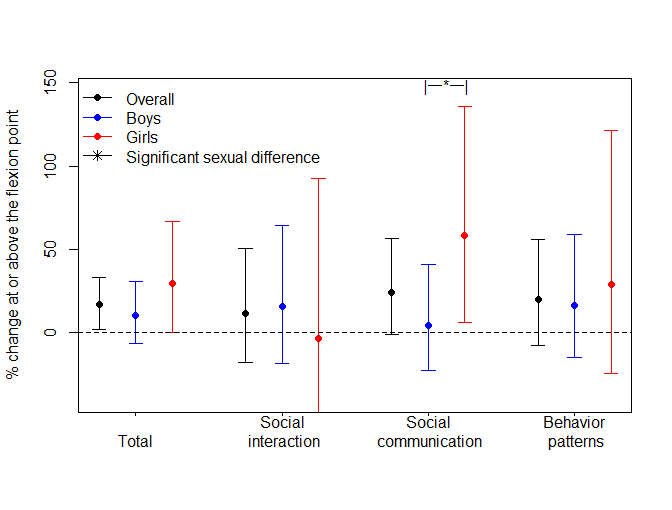


Abbreviations: K-SCQ, Korean version of the Social Communication Questionnaire; BPA, Bisphenol A

* Statistically significant at α=0.1; vertical lines represent confidence intervals.

Models were adjusted for gender (boy or girl), parity (1st vs. other), mother’s education (high school or lower vs. more than high school), birth weight (kg), use of plastic dishes in the microwave oven (yes or no), and postnatal BPA levels.
